# Optimization of Ultrasound-Assisted Extraction, Preliminary Characterization and In Vitro Antioxidant Activity of Polysaccharides from Green Pea Pods

**DOI:** 10.3390/foods5040078

**Published:** 2016-11-28

**Authors:** Maryam Jalili Safaryan, Ali Ganjloo, Mandana Bimakr, Soheila Zarringhalami

**Affiliations:** Department of Food Science and Technology, Faculty of Agriculture, University of Zanjan, Zanjan 45371-38791, Iran; maryamjalili44@yahoo.com (M.J.S.); mandanabimakr@yahoo.com (M.B.); zaringhalami@znu.ac.ir (S.Z.)

**Keywords:** green pea pod, polysaccharide, ultrasound-assisted extraction, antioxidant activity

## Abstract

In this study, ultrasound-assisted extraction of green pea pod polysaccharide (GPPP) was investigated and optimized using a central composite response surface design coupled with a numerical optimization technique. The effects of ultrasonic power (50–150 W), sonication time (20–80 min), ratio of water to raw material (20:1–40:1 mL/g) and extraction temperature (40–80 °C) on polysaccharide extraction yield were studied. The maximum extraction yield was obtained with a sonication power of 135.34 W, extraction time of 48.61 min, ratio of water to raw material of 33.6:1 mL/g and extraction temperature of 68.25 °C. Under these conditions, the experimental yield was 7.37% ± 0.13%, which was in close agreement with the predicted value (7.20%). The GPPP has been analyzed in order to identify a variety of chemical properties. The FT-IR spectrum demonstrated obvious characteristic peaks of polysaccharides. Furthermore, antioxidant activity of GPPP was evaluated by various antioxidant assays in vitro. The results revealed that GPPP possessed considerable DPPH free radical scavenging activity (91.03%), reducing power (0.63) and ferric reducing antioxidant power (0.34 mmol/L) at a total amount of 0.9 mg/mL. These findings indicated that GPPP extracted using an ultrasound-assisted extraction technique has potential as a novel source of natural antioxidant agent for future applications.

## 1. Introduction

The green pea (*Pisum sativum* L.) is one of the most important legumes in the world. It originated in the Middle East, and nowadays, it is cultivated in Europe and North America [1,2]. According to FAO statistics, the global green pea production in 2013 was about 18.5 million tons. The main producers of this product are China, India, the United States, France, and Algeria [3]. Green peas contain many nutrients such as protein, complex carbohydrates, dietary fiber, minerals, vitamins, and antioxidant compounds, and also are low in fat and have no cholesterol, so they have been used in the human diet for a long time [4]. Green pea pods contain an important amount of vegetable protein and a significant concentration of potassium and iron. The use of functional material extracted from this plant material as a food additive can be valuable to satisfy nutritional requirements due to the presence of large amounts dietary fiber and nutrients [5].

Nowadays, the disposal of agricultural food waste and by-products of food processing plants can be severe environmental issues. In this regard, green pea pods are considered a biological waste material, and their conversion into valuable compounds such as polysaccharides not only reduces the need for waste disposal but also provides an economically viable alternative resource for natural polysaccharide production.

Polysaccharides are high molecular weight macromolecules which can be easily separated from various sources such as plants, microorganisms and algae using water under appropriate conditions [6,7]. In recent years, studies on polysaccharide extraction from natural resources has gained increasing attention, as they can modulate rheological properties of foods and are generally used as natural food thickeners, gelling agents, coating agents, texture modifier, stabilizers, and emulsifiers for various applications [8].

Conventionally, plant polysaccharides are extracted by hot water procedures. Despite its simplicity and safety, hot water procedures generally require high temperatures and long processing time which may lead to the degradation of the polysaccharides and a decrease of biological activity of target compounds and also extraction yield is reduced due to destruction of polysaccharides [9,10,11]. Ultrasound-assisted extraction (UAE) has been successfully implemented in the extraction of polysaccharides from various plant material due to reducing solvent usage, lowering extraction temperature and enhanced extraction efficiency [12]. Recently, several studies have reported the possibility of using ultrasound technology in extraction of polysaccharide from food waste and their by-products including *Nephelium lappaceum* L. fruit peel [13] and pomegranate peel [14]. The mechanism of ultrasound-assisted extraction is attributed to cavitation, mechanical and thermal effects which can result in particle size reduction, disruption of cell walls and enhanced mass transfer across cell membranes [12]. Therefore, UAE provides increased extraction yield, increased rate of extraction, reduced extraction time and higher processing throughput along with the advantage of usage of reduced temperature and solvent volume which is very useful for the extraction of heat labile compounds [14].

On the other hand, use of an optimization technique such as response surface methodology (RSM) in the extraction processes can be very interesting because fewer experimental trails are needed compared to the single-factor method. Moreover, this technique can identify the interaction between different variables by establishing the appropriate mathematical model [13]. Central composite design (CCD) is one of the successful designs which applied for optimization of polysaccharides extraction process from various plant materials [15,16,17,18,19].

To the best of our knowledge, no reports are available on the optimization of ultrasound-assisted extraction of polysaccharides from green pea pods using RSM. Hence, an attempt was made for the first time to investigate and optimize the ultrasound-assisted extraction of polysaccharide from green pea pods using four independent variables including ultrasonic power (50–150 W), sonication time (20–80 min), ratio of water to raw material (20:1–40:1 mL/g), and extraction temperature (40–80 °C) through RSM. Then, chemical composition analysis, Fourier transform infrared (FT-IR) spectroscopy, and in vitro antioxidant activity assays were conducted to explore the potential of green pea pod as an alternative source of polysaccharide.

## 2. Materials and Methods

### 2.1. Materials

Fresh green pea pods were collected from the local processing unit near Zanjan, Iran. Green pea pods were washed with distilled water and were dried in the shade at room temperature until it attains constant weight. The dried green pea pods were ground into powder using a food grinder and passed through a standard 30-mesh sieve to obtain the homogeneous sample powder and then stored in the dark at −18 °C prior to extraction. All chemicals and solvents used in this study were of analytical grade (Sigma-Aldrich, St. Louis, MO, USA).

### 2.2. Polysaccharides Extraction

Dried green pea pod power was refluxed with 95% ethanol at 70 °C for 2 h to remove ethanol-soluble substances. Then the mixture was filtered through filter paper, and the residue was dried at 40 °C for 24 h.

The dried residue (4.0 g) was mixed with a specified amount of distilled water. The extraction process was performed using a 24 kHz ultrasonic device (UP200H—Hielscher, Teltow, Germany) with a 3.00 mm flat tip probe according to the experimental design (Table 1). The supernatant was separated from insoluble residue using filter paper under vacuum. The supernatant was centrifuged at 5000 rpm for 15 min, and then it was concentrated with a rotary evaporator. The concentrated supernatant was treated with the Sevag reagent [20] three times to remove protein. The concentrated supernatants were mixed with four volumes of 95% ethanol and kept overnight at 4 °C. Then the precipitates were collected by centrifugation (5000 rpm for 20 min), washed three times with ethanol and acetone to obtain the crude extract. Finally, the washed precipitates were dried at 40 °C until its weight was constant. The extraction yield of crude polysaccharide was calculated as follows:

$$Extraction\;yield\left(\%\right)=\frac{W_{2}}{W_{1}}\times 100$$

where *W*_2_ is the weight of dried crude polysaccharide (g), and *W*_1_ is the dried green pea pod powder weight (g).

### 2.3. Experimental Design, Optimization and Validation of Optimized Conditions

The extraction parameters were optimized through central composite response surface methodology. On the basis of preliminary results (data not shown), proper ranges of four independent variables including ultrasonic power (X_1_), sonication time (X_2_), ratio of water to raw material (X_3_) and extraction temperature (X_4_) were obtained, and then central composite design with three levels was performed. The extraction yield of crude polysaccharide (Y) was taken as the dependent variable of design experiment. As shown in Table 1, the whole design was composed of 30 experimental runs carried out in random order, with six replications at the center point to estimate the repeatability of the method. An empirical second-order polynomial model was fitted to correlate the response and independent variables as follows:

(1) $$Y\left(\%\right)=\beta_{o}+\overset{4}{\underset{i=1}{\sum }}\beta_{i}X_{i}+\overset{4}{\underset{i=1}{\sum }}\beta_{ii}X_{i}^{2}+\overset{4}{\underset{i<j=3}{\sum }}\beta_{ij}X_{i}X_{j}$$

where *Y* (%) is the dependent variable, and *β*_0_, *β_i_*, *β_ii_*, and *β_ij_* are the regression coefficients for model intercept, linear, quadratic, and interaction terms, respectively; while *X_i_* and *X_j_* are the independent variables.

Experimental design, data analysis, and quadratic model building were conducted using the Stat ease Design Expert 7.0.0 statistical software (Stat-Ease Inc., Minneapolis, MN, USA). The significance of all the terms in the model was assessed by the probability (*p*) of 0.05. The adequacy of the model was checked accounting for determination coefficient (*R*^2^), adjusted-*R*^2^, the coefficient of variance (C.V.%) and predicted error of sum of square (PRESS) [21].

Numerical optimization was carried out to predict the exact optimum level of independent variables leading to the desirable response goal. In this regard, all the independent variables were kept within range, while the response was maximized. Additional triplicate experiments were carried out under optimal extraction conditions in order to determine the validity of optimized conditions. The average value of the validation experiment was compared with the predicted value of the developed condition in order to find out the accuracy and suitability of the optimized conditions [21].

### 2.4. Characterization of GPPP

#### 2.4.1. Chemical Properties Analysis

The chemical properties of GPPP at optimal condition was evaluated in triplicate using the following methods: phenol–sulfuric acid test [22], Fehling’s test [23], Coomassie brilliant blue reaction [24]. The content of total polyphenols was estimated by the Folin–Ciocalteu colorimetric method [25], and it was expressed as mg gallic acid equivalent (GAE) per 100 mg sample.

#### 2.4.2. UV-Vis, FT-IR Spectroscopy

2 mg/mL aqueous solution of GPPP was prepared and scanned from 200 to 400 nm at 25 °C with a UV-Vis spectrophotometer (Specord 250, ANALYTIK JENA, Jena, Germany) to obtain its UV spectra.

The polysaccharide was ground with KBr powder and pressed into a pellet for Fourier-transform infrared (FT-IR) measurement. IR spectrum was recorded in the range of 4000–400 cm^−1^ on an FT-IR spectrometer [26].

#### 2.4.3. Determination of the Degree of Esterification

##### Titrimetric Method

The degree of esterification (DE) of green pea pod polysaccharide was determined by the titrimetric method of USP 26 NF 21 [27] and Food Chemical Codex [28] with slight modifications. 500 mg of dried crude polysaccharide was added to 20 mL of ethanol-HCl mixture (5 mL of 12 M HCl in 100 mL of 70% (*v*/*v*) ethanol) and vortexed for 10 min. Then the mixture filtered through filter paper. The collected filtrate was washed five times using the ethanol-HCl mixture as above (20 mL each time), and then washed with 70% (*v*/*v*) ethanol solution until the filtrate did not have chloride ions by silver nitrate detection. Finally, it was dried at 105 °C for 1 h. The dried samples (60–80 mg) were moistened with 2 mL of ethanol, dissolved in 100 mL of distilled water without carbon dioxide, and titrated by 0.1 M sodium hydroxide (NaOH) solution using phenolphthalein as indicator. The used volumes of NaOH were recorded as *V*_1_. Thereafter, 0.5 M NaOH was added into titrated sample solutions for demethoxylation. After 15 min, 0.5 M HCl, which was the same volume as that of 0.5 M NaOH used above, was added to titrated sample solution. The sample was titrated using 0.1 M NaOH, and the used volume was recorded as *V*_2_. DE was expressed as *V*_2_/(*V*_1_ + *V*_2_) × 100.

##### FT-IR Spectroscopic Method

The DE is defined as ratio of the area of the band at 1730 cm^−1^ (corresponding to the number of esterified carboxylic groups) over the sum of the areas of the bands at 1730 and 1600 cm^−1^ (corresponding to the number of total carboxylic groups). Therefore, the spectrum was recorded in the range 4000 to 400 cm^−1^ and the *DE* was determined using the following equation:

$$DE=\frac{A_{1730}}{A_{1730}+A_{1600}}\times 100$$

where *A*_1730_ was defined as the area of the band at 1730 cm^−1^ and *A*_1600_ was defined as the area of the band at 1600 cm^−1^.

### 2.5. Determination of In Vitro Antioxidant Activity

#### 2.5.1. Assay of DPPH Free Radical Scavenging Activity

The 2,2-diphenyl-1-picrylhydrazyl (DPPH) radical scavenging activity of green pea pod polysaccharide was determined according to the method described by Xie et al. [29] with some modifications. Briefly 2.0 mL of various concentrations (0.1, 0.3, 0.5, 0.7 and 0.9 mg/mL) of the polysaccharide was thoroughly mixed with 2.0 mL of DPPH solution (0.1 mM DPPH in 95% ethanol). The mixture was shaken vigorously and kept in the dark for 30 min at room temperature. The absorbance was measured at 517 nm with a UV-vis spectrophotometer. The lower absorbance of the reaction mixture indicates higher free radical scavenging activity. 95% ethanol was used as the blank control, and ascorbic acid was used as a positive control activity. The capability of polysaccharide to scavenge the DPPH free radical was calculated according to the following equation:

(2) $$DPPH\;free\;radical\;Scavenging\;activity\;\left(\%\right)=\frac{1-(A_{2}-A_{1})}{A_{0}}\times 100$$

where *A*_0_ is the absorbance of the DPPH solution without addition of the sample or positive control, *A*_1_ is the absorbance of the sample without DPPH solution, and *A*_2_ is the absorbance of the incubation mixture containing both the sample and DPPH solution.

#### 2.5.2. Determination of Reducing Power

The reducing power of green pea pod polysaccharide was determined according to the method described by Oyaizu [30]. Briefly, various concentrations of polysaccharide (0.1–0.9 mg/mL, 2.0 mL) were mixed with 2.0 mL phosphate buffer (0.2 M, pH 6.6) and 2.0 mL potassium ferricyanide (K_3_Fe(CN)_6_) (1%). The mixture was incubated at 50 °C for 20 min. When the samples cooled to room temperature, 2.0 mL trichloroacetic acid (10%, *w*/*v*) was added to the reaction mixture. Afterward, the mixture was centrifuged at 3000 rpm for 10 min. Subsequently, 2.0 mL of the upper layer was mixed with 2.0 mL distilled water and 0.4 mL of ferric chloride (FeCl_3_, 0.1% in *w*/*v*) in a test tube. After 10 min reaction at 50 °C, the absorbance was measured at 700 nm. The higher absorbance of the reaction mixture indicated greater reducing power. Ascorbic acid was used as a positive control.

#### 2.5.3. Ferric Reducing Antioxidant Power Assay (FRAP)

The ferric reducing antioxidant power (FRAP) assay was carried out according to the method of Benzie and Strain [31]. The assay was carried out according to Benzie and Strain method. FRAP reagent was prepared adding 0.3 mol/L acetate buffer (pH 3.6), 10 mmol TPTZ solution in 40 mmol HCL and 20 mmol iron (III) chloride solution in proportions of 10:1:1 (*v*/*v*), respectively. The reagent was warmed at 37 °C prior to use. 50 µL of different extracts were added to 1.5 mL of the FRAP reagent. Absorbance of the reaction mixture was recorded at 593 nm using UV-vis spectrometer after 4 min against blank. A standard calibration curve was prepared using various concentration of FeSO_4_.

## 3. Results and Discussion

### 3.1. Model Fitting and Statistical Analysis

The effect of independent variables including ultrasonic power, sonication time, extraction temperature and ratio of water to raw material and their combinations on the extraction yield of polysaccharide from green pea pod was studied and optimized through central composite response surface methodology. The extraction yield of GPPP was varied from 4.45% to 7.08% (Table 1).

Analysis of variance (ANOVA) was used to analyze the experimental data and the results are listed in Table 2. The most of the variation in the response could be explained through the regression equation due to the high model *F*-value (321.03 for polysaccharide extraction yield) and the associated low *p*-value (*p* < 0.0001) which indicated the significance of developed model. The accuracy and good correlation between the response and the independent variables were clearly demonstrated by the coefficient of determination (*R*^2^) and adjusted coefficient of determination (adj-*R*^2^). In this model, the *R*^2^ and adj-*R*^2^ values were 0.996 and 0.993, respectively. Reproducibility and good reliability of the model was confirmed by the low value of coefficient of variance (0.97) [30]. In this study, the adequate precision (signal-to-noise ratio) was found to be >61 for the response, which indicates the best fitness of the developed model. The synergistic effect of the independent variables on the extraction yield was indicated by a positive sign of the coefficients, whereas an antagonistic effect was indicated by negative coefficients [32].

A second-order polynomial model (Equation (3)) was generated by applying multiple regression analysis to the experimental data to express the relationship between the independent variables and the response. The final equation obtained in terms of coded factors is given as follows:

(3) $$Extraction\;yield\left(\%\right)=\;6.91+0.34X_{1}+0.25X_{2}+0.35X_{3}+0.37X_{4}+0.076X_{1}X_{2}+0.033X_{1}X_{3}+0.11X_{1}X_{4}-0.13X_{2}X_{3}-0.047X_{2}X_{4}-0.054X_{3}X_{4}-0.31X_{1}^{2}-0.20X_{2}^{2}-0.34X_{3}^{2}-0.30X_{4}^{2}$$

where *X_1_*, *X_2_*, *X_3_* and *X_4_* are the coded variables for the ultrasonic power (W), sonication time (min), ratio of water to raw material (mL/g) and extraction temperature (°C), respectively. 

### 3.2. Analysis of Response Surfaces, Optimization of Extraction Parameters and Validation of the Predictive Model

**Figure 1:**
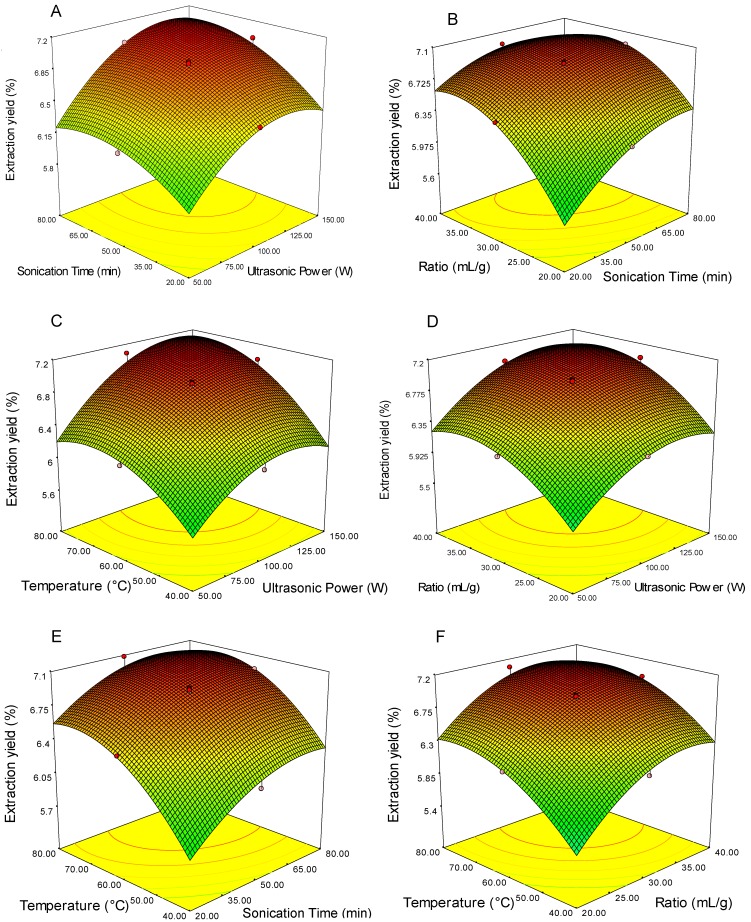
Response surface plots showing the effects of independent variables on the extraction yield of GPPP: (**A**) Extraction yield vs. sonication time (min) and ultrasonic power (W); (**B**) Extraction yield vs. ratio (mL/g) and sonication time (min); (**C**) Extraction yield vs. ultrasonic power (W) and temperature (°C); (**D**) Extraction yield vs. ratio (mL/g) and ultrasonic power (W); (**E**) Extraction yield vs. temperature (°C) and sonication time (min); (**F**) Extraction yield vs. temperature (°C) and ratio (mL/g).

Three-dimensional (3D) response surface plots were generated to visualize the effects of experimental levels of each variable on the response, the type of interactions between of two independent variables, and to determine the optimum level of each independent variables for maximum extraction yield of GPPP. In each plot, the interaction of two independent variables was investigated simultaneously, while the others were fixed constant at zero level. The 3D response surface plots were presented in Figure 1A–F.

Ultrasonic power is one of the most important parameters affecting the extraction yield of polysaccharide and cost of the process especially at industrial scale. The extraction yield of GPPP as a function of sonication time and ultrasonic power at fixed extraction temperature (60 °C) and ratio of water to raw material (30 mL/g) is given in Figure 1A. It can be seen that extraction yield of GPPP increased with increasing sonication time up to 35 min, and the extraction yield of GPPP was also found to increase with the increase of ultrasonic power up to 100 W. The higher extraction yield at high sonication power could be attributed to different mechanisms such as the creation and collapse of more bubbles resulting in swelling of the materials, solvent uptake and enlargement of the pores in the materials and washing out of cell content due to generation of strong liquid microjets and acoustic streaming rupturing the cells [33,34]. The extraction yield was no longer changed when the ultrasound power exceeded 100 W. These results could be attributed to the intensive cavitation, turbulence shear, and instantaneous high pressure drop involved in processing. With the increase in ultrasonic intensity and treatment time, the ultrasonic energy was sufficient to break down the strong bonds, such as glycosidic linkages, connecting the sugar units. It was pointed out that intense ultrasonic power would result in degradation and decomposition of the polysaccharide [35,36]. This result was in accordance with other reports for polysaccharide extraction from various raw materials [8,33,37].

Generally, a long extraction time favored polysaccharide production. On the other hand, long extraction time may induce decomposing and structural destruction of polysaccharides [37]. The extraction yield of GPPP at varying sonication times and ratios of water to raw material at fixed extraction temperature (60 °C) and ultrasonic power (100 W) is shown in Figure 1B. It is obvious that the extraction yield of GPPP increased within the ratio of water to raw material up to 35 mL/g and the extraction yield increased with the increase of the extraction time up to 60 min. Ultrasonic waves facilitated the release of polysaccharides through ruptured cells at the early period of extraction. The current finding seems to be consistent with other researches [8,33,38].

Usually, in the solid–liquid extraction process, the higher temperature could enhance the recovery of target compounds from raw materials. However, the degradation reaction was also reported. The 3D response surface plot at varying extraction temperature and ultrasonic power at fixed sonication time (50 min) and ratio of water to raw material (30 mL/g) is shown in Figure 1C. The extraction yield of GPPP increased with increasing the extraction temperature up to 65 °C, and no further changes were observed beyond the temperature of 70 °C while the extraction yield of GPPP increased with the increase of the ultrasonic power to 100 W. It was pointed out that the solvent viscosity decreases and the cavitation and vapor pressure of solvent increases within the tissue with increasing temperature (up to 65 °C) so that the area of contact between the particles and solvent is maximized [39]. Moreover, the mass transfer increased with increasing extraction temperature due to the increase of the polysaccharide solubility [40]. Therefore, increasing the temperature leads to an increase in the extraction yield. However, high temperature led to the decrease of surface tension and the increase of vapor pressure within micro bubbles, causing the damping of the ultrasonic wave. This tendency is in agreement with other reports in extracting polysaccharides [8,33].

The ratio of water to raw material is one of the most important parameters from economical point of view. Generally, a larger ratio of water to raw material can dissolve target compounds more effectively. 3D response surface plot at varying ultrasonic power and ratio of water to raw material ratio at fixed sonication time (50 min) and extraction temperature (60 °C) is shown in Figure 1D. It can be seen that extraction yield of GPPP increased with increase of sonication power from 50 to 100 W, then did not further increase with increasing sonication power, and reached the maximum value when ratio of water to raw material at 30 mL/g, and beyond this level, extraction yield of GPPP was constant.

The contact area between material and water increased with increasing ratio of water to raw material, fully dissolving the polysaccharide from the material and leading to higher diffusion, but distribution of ultrasonic energy density in the extraction solution decreased at a higher ratio (>30:1 mL/g), resulting in no obvious change in the extraction yield of polysaccharide from 30:1 to 40:1 mL/g. Moreover, it is pointed out that the distance of diffusion towards the interior tissues was prolonged at a high ratio of water to raw material [36]. The same tendency was also reported for polysaccharide extraction from mulberry leaves [36] and *Nephelium lappaceum* L. fruit peel [13].

Figure 1E shows the 3D response surface plot at varying extraction temperatures and extraction times at fixed sonication power (100 W) and ratio of water to raw material (30 mL/g). The extraction yield of GPP increased within the extraction temperature from 40 to 60 °C, and the extraction yield also increased with the increase of the extraction time from 20 to 50 min, and beyond these levels did not further increase.

Finally, a 3D response surface plot at varying extraction temperatures and ratios of water to raw material at fixed sonication power (100 min) and extraction time (50 min) is shown in Figure 1F. The result showed that extraction yield of GPPP increased with the increasing of extraction temperature from 40 to 60 °C, but beyond this level, extraction yield of GPPP was constant with the increase of extraction temperature. Furthermore, extraction yield of GPPP was also increased when the ratio of water to raw material was increased in the range from 20 to 30 mL/g. The result of numerical optimization showed that the optimized conditions were ultrasonic power of 135.34 W, extraction time of 48.61 min, ratio of water to raw material 33.6:1 mL/g and extraction temperature of 68.25 °C. Under these conditions, the maximum extraction yield predicted by the model was 7.20%. However, considering the operability in actual production, the optimal conditions can be modified as follows: ultrasonic power of 135 W, sonication time of 50 min, ratio of water to raw material of 30 mL/g and extraction temperature of 68 °C. Triplicate experiments at the modified optimal extraction conditions were carried out to compare the predicted results with the experimental value. Under the modified conditions, the experimental yield of GPPP was 7.37% ± 0.13%, which was close to the predicted value (7.20%). As a result, central composite design was found to be an accurate and decisive tool for predicting the extraction yield of polysaccharides from green pea pods using an ultrasound-assisted extraction technique.

### 3.3. Preliminary Characterization of GPPP

**Figure 2:**
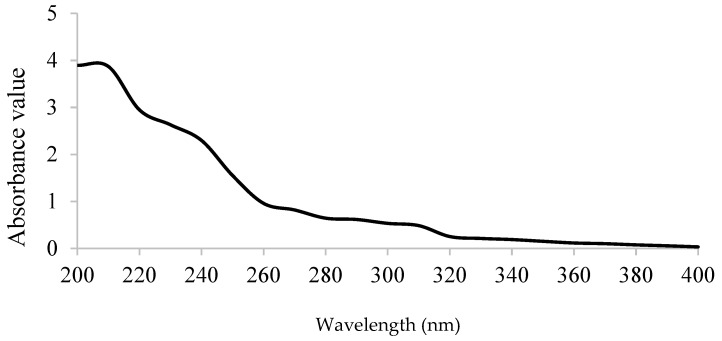
UV-vis spectrum of GPPP in the range of 200–400 nm.

The chemical composition of GPPP extracted under optimized conditions was determined. The total carbohydrate content of GPPP was 66.4% ± 1.16%, which indicated that it contained high levels of carbohydrates. The uronic acid content of GPPP was 9.22% ± 0.89%. No protein was detected in GPPP, which indicated a high purity of the extracted polysaccharide. Therefore, ~25% remaining could be related to insoluble phenolics, free sugars or other compounds bound to cell wall components or sulfuric radicals. Furthermore, there was also no absorption at 260–280 nm in the UV-vis spectrum (Figure 2), indicating the absence of nucleic acid and protein, respectively [6]. Fehling test was negative for GPPP. The total phenolic content of GPPP was 0.17 ± 0.01 mg GAE/100 mg sample, indicating that the antioxidant activity was mainly attributed to the GPPP.

### 3.4. FT-IR Spectroscopy

**Figure 3:**
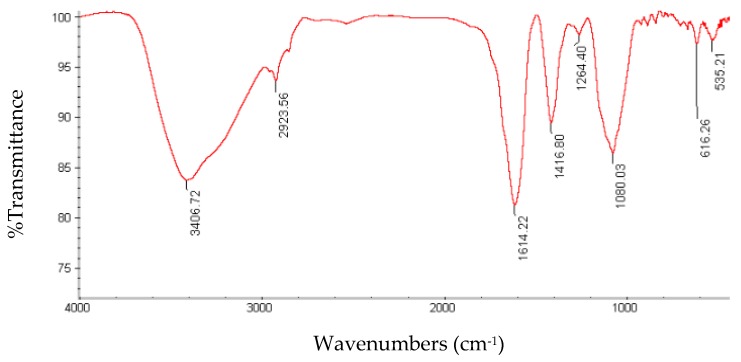
FT-IR spectrum of GPPP at optimum conditions.

FT-IR spectroscopy can be used for approximate identification of polysaccharides in plant materials when combined with chemical analyses [41]. The FT-IR spectrum of GPPP at modified optimal conditions is shown in Figure 3. The strong peak of the O–H stretching vibration was observed at 3406.72 cm^−1^, and the weak absorption peak of the C–H stretching vibration was found at 2923.56 cm^−1^, including CH, CH_2_ and CH_3_ stretching and bending vibrations. The C=O asymmetric stretching vibrations of the carboxylate (–COO^−^) groups was observed at 1614.22 cm^−1^. The absorption peak at around 1416.22 indicated the presence of a carboxyl group. The peaks in the range 1300–1000 cm^−1^ were characteristic of carbohydrates. The peaks around 1264 cm^−1^ and 1086 cm^−1^ were caused using the stretching vibration of C–O–C on the glycosidic ring and C–OH, respectively [6]. The absorption bands at about 590–900 cm^−1^ were attributed to skeletal modes of pyranose, and this FT-IR analysis result confirmed that the monosaccharide composition of green pea polysaccharides mainly contains pyranose [42]. The peaks in the range 330–960 cm^−1^ were characteristic of acidic pectins, xyloglucans and xylogalacturonans.

### 3.5. The Degree of Esterification of GPPP

The DE value obtained by titrimetric method was compared against the value obtained from the FT-IR method (Table 3) in order to evaluate the validation of the titrimetric method. The results confirmed that no significant difference observed between the DE from the two methods.

### 3.6. DPPH Radical Scavenging Activity of GPPP

**Figure 4:**
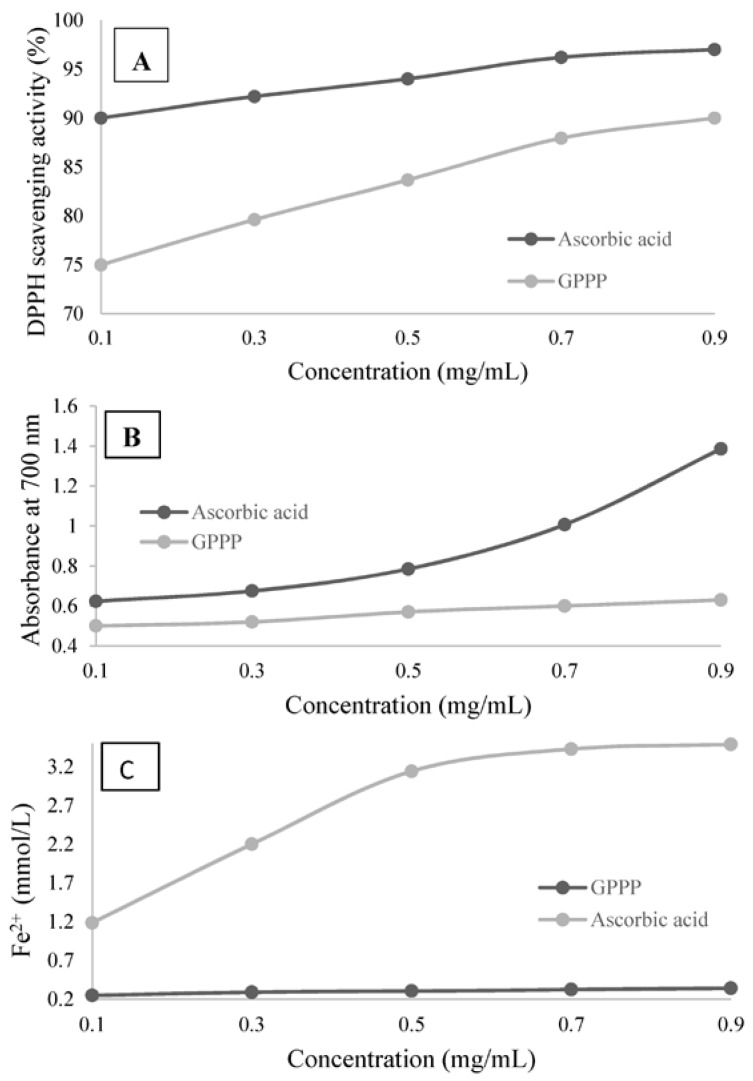
Antioxidant activities of GPPP and ascorbic acid: (**A**) DPPH free radical scavenging activity; (**B**) reducing power and (**C**) FRAP.

DPPH^•^ is a stable free radical which has been widely used to estimate the free radical scavenging ability of antioxidants [16]. The DPPH free radical is a stable radical with a maximum absorption at 517 nm that can readily undergo scavenging by an antioxidant. The principle of DPPH analysis is based on the color change of the solution from purple to yellow as radicals are quenched by antioxidant compounds, which can be quantitatively measured from the changes in absorbance [23]. On the basis of this principle, the antioxidant activity of polysaccharides can be expressed as their ability to scavenge DPPH free radicals. The scavenging activity of GPPP on the DPPH^•^ radicals is shown in Figure 4A and compared with ascorbic acid as a positive control in a concentration-dependent manner. The DPPH^•^ radical scavenging activity of GPPP increased at the concentration range (0.1–0.9 mg/mL) of the sample from 76.55% to 91.03%. The highest scavenging activity of GPPP was 91.03% ± 1.54% at 0.9 mg/mL. However, scavenging activity of ascorbic acid was 95.2% ± 1.7% at the same concentration. Therefore, it is clear that GPPP had a noticeable effect on scavenging DPPH^•^ free radicals, especially at high concentrations. The possible mechanism by which GPPP acts as an antioxidant may be attributed to the existence of numerous hydroxyls in a polysaccharide molecule, which could provide electron or hydrogen donation power to the free DPPH radicals and neutralize them [43].

### 3.7. Reducing Power

The reducing power assay based on the reduction of Fe^3+^ to Fe^2+^ by donating an electron could be used as an important indicator to evaluate the potential antioxidant activity of polysaccharides [44]. A direct correlation between the antioxidant and reducing capacity has been reported. Reducing properties are generally associated with the presence of reductones, which can donate a hydrogen atom and exert antioxidant action by breaking the free-radical chain. Reductones are also reported to react with certain precursors of peroxide, preventing peroxide formation. Thus, the reducing capacity of a compound or extract may be a significant indicator of its potential antioxidant activity. According to the reports which claimed that the reducing power is positively related to the antioxidant activities [45], the antioxidant capacity of GPPP was evaluated using the K_3_Fe(CN)_6_ reduction method at 700 nm. The reducing power of GPPP and ascorbic acid was shown in Figure 4B. A higher absorbance value means greater reducing ability of samples. The reducing power of GPPP was increased from 0.5 to 0.63 in the range of 0.1–0.9 mg/mL. It is clear that the reducing power of GPPP correlated well with sample concentrations. According to Figure 4B, the reducing power of GPPP was significantly lower than that of ascorbic acid. It was revealed that the reducing power was generally associated with the presence of reductones. Therefore, this result suggested that GPPP might contain reductone-associated and hydroxyl groups [15,46]. The data of reducing power suggested that GPPP could act as electron donors and can react with free radicals to convert them to more stable products [47].

### 3.8. Ferric Reducing Antioxidant Power (FRAP)

Since the antioxidant activity of a substance is correlated directly to its reducing capacity, the FRAP assay can be applied to study its antioxidant capacity. Thus, the antioxidant activity of GPPP is evaluated via the ability to reduce the Fe^3+^-TPTZ complex to the Fe^2+^-TPTZ complex [48]. The antioxidant capacities of GPPP and ascorbic acid are shown in Figure 4C. In the range of 0.1–0.9 mg/mL for UAE, the FRAP values were increased from 0.25 to 0.34 mmol/L. The antioxidant capacity of GPPP correlated well with increasing concentration. However, compared with ascorbic acid, the antioxidant capacity of GPPP was weak.

## 4. Conclusions

In this study, the ultrasound-assisted extraction of GPPP has been optimized by applying three-level, four-factor central composite response surface methodology. The optimum conditions for ultrasound-assisted extraction of GPPP were found to be ultrasonic power of 135.34 W, sonication time of 48.61 min, ratio of water to raw material of 33.6:1 mL/g and extraction temperature of 68.25 °C. Under these conditions, the experimental yield of GPPP was very close to the predicted yield value. The FT-IR spectrum demonstrated obvious characteristic peaks of polysaccharides. Moreover, the polysaccharides exhibited high reducing power and free-radical scavenging activities in vitro. The overall findings indicate that polysaccharides from a low-cost resource such as green pea pods have great potential to be developed as natural antioxidants for use in functional foods and medicine. Further studies on purification, structure and evaluation of antioxidant activity in vivo are in progress.

## Figures and Tables

**Table 1:** Central composite design and response value for the extraction yield of green pea pod.

Run Order	Uncoded Variable Levels				Extraction Yield (%)	
	X_1_: Ultrasonic Power (W)	X_2_: Sonication Time (min)	X_3_: Ratio of Water to Raw Material (mL/g)	X_4_: Extraction Temperature (°C)	Experimental	Predicted
1	50.00	20.00	20.00	40.00	4.45	4.41
2	150.00	20.00	20.00	40.00	4.70	4.66
3	50.00	80.00	20.00	40.00	5.17	5.12
4	150.00	80.00	20.00	40.00	5.60	5.67
5	50.00	20.00	40.00	40.00	5.40	5.43
6	150.00	20.00	40.00	40.00	5.80	5.81
7	50.00	80.00	40.00	40.00	5.62	5.60
8	150.00	80.00	40.00	40.00	6.34	6.29
9	50.00	20.00	20.00	80.00	5.10	5.14
10	150.00	20.00	20.00	80.00	5.80	5.83
11	50.00	80.00	20.00	80.00	5.65	5.66
12	150.00	80.00	20.00	80.00	6.70	6.66
13	50.00	20.00	40.00	80.00	6.00	5.94
14	150.00	20.00	40.00	80.00	6.73	6.77
15	50.00	80.00	40.00	80.00	5.90	5.93
16	150.00	80.00	40.00	80.00	7.00	7.05
17	50.00	50.00	30.00	60.00	6.20	6.25
18	150.00	50.00	30.00	60.00	7.00	6.94
19	100.00	20.00	30.00	60.00	6.47	6.45
20	100.00	80.00	30.00	60.00	6.95	6.95
21	100.00	50.00	20.00	60.00	6.20	6.21
22	100.00	50.00	40.00	60.00	6.95	6.92
23	100.00	50.00	30.00	40.00	6.15	6.23
24	100.00	50.00	30.00	80.00	7.08	6.98
25	100.00	50.00	30.00	60.00	6.91	6.91
26	100.00	50.00	30.00	60.00	6.93	6.91
27	100.00	50.00	30.00	60.00	6.88	6.91
28	100.00	50.00	30.00	60.00	6.88	6.91
29	100.00	50.00	30.00	60.00	6.92	6.91
30	100.00	50.00	30.00	60.00	6.89	6.91

**Table 2:** Analysis of variance (ANOVA) for regression model of extraction yield.

Source	Sum of Squares	Degree of Freedom	Mean Squares	*F*-Value	*p*-Value
Model	16.37	14	1.17	321.03	<0.0001
X_1_-Ultrasonic power	2.12	1	2.12	582.38	<0.0001
X_2_-Sonication time	1.12	1	1.12	306.04	<0.0001
X_3_-ratio of water to raw material	2.25	1	2.25	618.74	<0.0001
X_4_-Extraction temperature	2.52	1	2.52	690.65	<0.0001
X_1_ X_2_	0.09	1	0.09	25.53	<0.0001
X_1_ X_3_	0.01	1	0.01	4.64	0.0479
X_1_ X_4_	0.20	1	0.20	54.35	<0.0001
X_2_ X_3_	0.29	1	0.29	78.56	<0.0001
X_2_ X_4_	0.03	1	0.03	9.91	0.0066
X_3_ X_4_	0.04	1	0.04	12.69	0.0028
Residual	0.05	15	3.64 × 10^−3^	11.74	
Std. dev.	0.06	*R*^2^		0.99	
Mean	6.21	Adj-*R*^2^		0.99	
CV%	0.97	Pred-*R*^2^		0.97	
PRESS Signal-to-noise ratio	0.33 >61	Adequate precision		61.85	

**Table 3:** The degree of esterification of GPPP obtained from different methods.

Method	Degree of Esterification (%)
Titrimetric method	53.28 ± 0.08
FT-IR spectroscopy	51.36 ± 0.03
